# Bacterial Pathogen Identification and Its Association with Clinical, Ultrasonographic, and Post-Mortem Severity in Lacaune Lambs with Ovine Respiratory Complex

**DOI:** 10.3390/ani16131985

**Published:** 2026-06-27

**Authors:** Alejandro Sánchez-Fernández, Alejandro de la Peña-Moctezuma, Begoña Álvarez, Francisco Revert-Ros, Marta González Clari, Juan Carlos Gardón, Joel Bueso-Ródenas

**Affiliations:** 1 Doctoral School, Catholic University of Valencia San Vicente Mártir, 46001 Valencia, Spain; alejandro.sanchez@ucv.es; 2 Center for Teaching, Research and Extension in Animal Production in the Highlands, Faculty of Veterinary Medicine and Zootechnics, National Autonomous University of Mexico, Mexico City 04510, Mexico; delapema@unam.mx; 3 Veterinary and Experimental Science Faculty, Catholic University of Valencia San Vicente Mártir, 46001 Valencia, Spain; begona.alvarez@ucv.es; 4 Department of Biomedical Sciences, School of Medicine and Health Sciences, Catholic University of Valencia San Vicente Mártir, 46001 Valencia, Spain; fj.revert@ucv.es; 5 Department of Animal Production and Public Health, Faculty of Veterinary Medicine and Experimental Sciences, Catholic University of Valencia San Vicente Mártir, 46001 Valencia, Spain; marta.gonzalez@ucv.es; 6 Department of Medicine and Animal Surgery, Faculty of Veterinary Medicine and Experimental Sciences, Catholic University of Valencia San Vicente Mártir, 46001 Valencia, Spain

**Keywords:** thoracic ultrasonography, respiratory disease, diagnosis, isolation, ovine, bacterial pathogens, *Mycoplasma ovipneumoniae*, *Pasteurella multocida*

## Abstract

Ovine Respiratory Complex is a multifactorial disease involving interactions between pathogens, host factors, and environmental conditions. This study investigated associations between selected bacterial agents, including *Mycoplasma ovipneumoniae* infection and members of the *Pasteurellaceae* family, and clinical, auscultatory, ultrasonographic, and post-mortem findings in Lacaune fattening lambs. A total of 80 animals were evaluated using clinical examination, thoracic auscultation, ultrasonography, post-mortem assessment, bacteriological culture, and quantitative PCR (qPCR) for *Mycoplasma ovipneumoniae*. Associations between microbial detection and disease severity scores were analyzed. *Mycoplasma ovipneumoniae* was the most frequently detected organism (50%), followed by *Pasteurella multocida* (32.9%) and *Trueperella pyogenes* (27.8%). Detection of specific microorganisms was more frequently observed in animals with increased disease severity across multiple diagnostic modalities. Coinfection was identified in 60.8% of animals and was significantly associated with increased severity according to clinical, auscultatory, ultrasonographic, and post-mortem scores. These findings underscore the polymicrobial nature of ORC and emphasise the necessity of employing complementary diagnostic approaches for disease evaluation.

## Simple Summary

Respiratory disease is one of the main health problems affecting lamb production worldwide, causing economic losses due to poor growth, treatment costs, and deaths. These diseases are often caused by several microorganisms acting together, making diagnosis and control difficult. This study investigated the relationship between different bacteria and the severity of respiratory disease in fattening lambs. Eighty lambs were examined using several methods, including clinical examination, lung auscultation, ultrasound imaging, post-mortem evaluation, bacterial culture, and molecular testing. *Mycoplasma ovipneumoniae* was the most frequently detected microorganism, followed by *Pasteurella multocida* and *Trueperella pyogenes*. Lambs carrying these microorganisms, especially when more than one bacterium was present at the same time, showed more severe respiratory disease and more extensive lung damage. The results confirm that respiratory disease in lambs is usually caused by multiple infectious agents acting together rather than by a single pathogen. The study also highlights the usefulness of combining different diagnostic techniques, particularly lung ultrasound and laboratory testing, to improve the detection and evaluation of respiratory disease in sheep production systems. Animals with higher amounts of bacterial genetic material also tended to present more severe lesions. Future studies should incorporate larger sample sizes, longitudinal follow-up, and viral diagnostics to further clarify the role of individual pathogens in disease progression and to validate ultrasonographic screening as a tool for targeted intervention in sheep production systems.

## 1. Introduction

Respiratory diseases constitute one of the most significant challenges in small ruminant production systems, resulting in substantial economic losses attributed to mortality, reduced weight gain, elevated treatment costs, and carcass condemnation at slaughterhouses [1,2]. The Ovine Respiratory Complex (ORC) is a multifactorial respiratory syndrome arising from complex interactions among infectious agents, host factors, and environmental stressors [3,4]. Young lambs during the fattening period are particularly susceptible, with prevalence rates ranging from 30% to 70% in affected flocks and mortality rates reaching 30–40% in severely affected animals [3,5,6].

Accurate diagnosis of ORC in field conditions requires multiple complementary approaches, each providing distinct information regarding disease presence, severity, and extent. Clinical evaluation based on respiratory signs, including coughing, nasal discharge, dyspnea, and fever, provides the initial indication of respiratory disease but has limited sensitivity for detecting subclinical pulmonary lesions or determining the precise extent of pulmonary involvement [7]. Thoracic ultrasonography (TUS) has emerged as a valuable non-invasive tool for evaluating pulmonary lesions in small ruminants [7,8,9], enabling visualization of consolidation patterns, pleural effusion, and B-lines in live animals [7,8,9,10,11]. Recent studies conducted in lambs and calves demonstrated strong correlations between ultrasonographic findings and both clinical scoring and post-mortem macroscopic evaluation [7,12], supporting the superior sensitivity of this imaging modality for detecting subclinical lesions compared to conventional auscultation [7]. Unlike auscultation, which detects only sound transmission abnormalities, TUS directly visualizes changes in lung tissue acoustic impedance caused by alveolar fluid accumulation and consolidation, pathological processes that may be present before clinical or auscultatory signs become detectable, thereby enabling identification of subclinical pulmonary involvement [7,8]. Post-mortem examination remains the gold standard for definitive diagnosis, permitting direct visualization, localization, and characterization of pulmonary lesions [13,14].

The bacterial etiology of ORC involves multiple pathogens within the *Pasteurellaceae* [13,15] and *Mycoplasmataceae* families [13,14,15,16] and opportunistic microorganisms [16]. *Mannheimia haemolytica* has traditionally been recognized as the primary agent of ORC [15,17], producing a potent leukotoxin that induces neutrophil apoptosis and pulmonary inflammation [18,19]. *Pasteurella multocida* is another major pathogen frequently isolated from pneumonic lungs, often in coinfection with *Mannheimia haemolytica* [20]. Additional pathogens, including *Bibersteinia trehalosi* [15], *Escherichia coli*, *Trueperella pyogenes*, Staphylococcus spp., Streptococcus spp., and Pseudomonas spp. [16] have been increasingly identified as contributors to the polymicrobial nature of ORC [14,21]. Importantly, many of these bacteria constitute the commensal microbiota of the upper respiratory tract in healthy sheep [22], with their transition from commensal organisms to pathobionts occurring when host defense mechanisms are compromised by stressful factors or viral infections [3]. Viral pathogens, including Parainfluenza-3 virus, Respiratory Syncytial Virus, and Ovine Herpesvirus-2, are recognized as primary insults that impair mucosal defenses and facilitate secondary bacterial colonization, with bacteria being the principal drivers of pulmonary lesion development in ORC.

A critical knowledge gap remains regarding whether specific etiological agents are associated with distinct lesion patterns across diagnostic modalities. Establishing these associations is biologically relevant because different pathogens employ distinct virulence mechanisms, potentially producing characteristic patterns of pulmonary injury detectable by ultrasonography and post-mortem examination. Understanding these relationships could improve diagnostic accuracy, enable pathogen-targeted therapeutic decisions, and support risk stratification in field conditions. Semiquantitative culture and quantitative PCR provide complementary tools for assessing pathogen load and its relationship with disease severity [23,24]. The present study aimed to determine whether detection of selected bacterial pathogens, particularly members of the *Pasteurellaceae* and *Mycoplasmataceae* families, was independently associated with increased severity across clinical (SClinic), auscultatory (SAusc), ultrasonographic (SUlt), and post-mortem (SPost) scoring systems in Lacaune fattening lambs with naturally occurring ORC, while accounting for pathogen co-occurrence and coinfection status.

## 2. Materials and Methods

### 2.1. Animals and Study Design

The study was conducted on a commercial dairy sheep farm rearing Lacaune sheep located in Catadau, the Valencian Community, Spain. Eighty Lacaune lambs (45 males and 35 females) from a batch of 840 animals destined for fattening were selected, with a mean age of approximately 70 days and a mean body weight of approximately 28 kg at slaughter. Lambs were separated from their dams at birth and transferred to artificial rearing facilities, where they received commercial milk replacer (ELVOR 63; 24% crude protein, 24% fat, 5% fiber, 7% ash, 0.9% calcium, 0.45% sodium, 0.75% phosphorus), cereal straw, and starter concentrate (Lactoiniciacor Nanta; 18% crude protein, 4% crude fiber, 3% fat, 6.9% ash, 43% starch and sugars). Weaning was performed at 40 days of age when lambs reached approximately 17 kg body weight. Following weaning, animals were transferred to fattening facilities until 70 days of age in 3 m × 13 m pens with approximately 40 animals per pen, with ad libitum access to cereal straw and fattening concentrate (Nantacor Intensive Fattening Nanta; 17.5% crude protein, 4.3% fiber, 3% fat, 42% starch and sugars).

All animal selection and diagnostic assessments were performed 12 h before transport to the abattoir. This cross-sectional study was conducted in a convenience-selected cohort of fattening lambs subjected to routine veterinary evaluation due to the presence of clinical conditions potentially associated with impaired productive performance, including respiratory, enteric, locomotor disorders, or growth retardation.

Lambs were not randomly selected from the general fattening population; instead, the study population was intentionally enriched for clinically affected individuals to investigate the association between respiratory pathogens and clinical, ultrasonographic, and post-mortem findings under field conditions.

Inclusion criteria comprised the presence of at least one of the following clinical or productive abnormalities: coughing, nasal discharge (serous, mucous, or purulent), ocular discharge, increased rectal temperature (≥39.5 °C), dyspnoea, reduced weight gain below the expected batch average, lethargy, or poor body condition score relative to age. Animals lacking sufficient clinical information or adequate post-mortem sampling were excluded from the study. Consequently, the findings should not be interpreted as representative prevalence estimates for the broader fattening lamb population. Although all selected animals presented at least one clinical or productive abnormality, the observed signs were mild to moderate in nature and did not meet the criteria for unfitness for transport established under Council Regulation (EC) No 1/2005 on the protection of animals during transport. Animals showing severe suffering, inability to stand unaided, or open wounds were excluded from transport and therefore from the study. Samples for gross pathological examination were collected.

### 2.2. Diagnostic Assessments and Scoring System

Clinical examination was performed using the clinical score (SClinic) adapted for lambs [7], evaluating ocular discharge (OD), nasal discharge (ND), head tilting (HT), cough, and rectal temperature (RT). Ocular and nasal discharges were classified as serous, mucous, or purulent, and as unilateral or bilateral. Cough was defined as a forceful and audible expiration. Rectal temperature was considered abnormal at ≥39.5 °C. Each parameter was scored from 0 (normal) to 3 (most severe), with total clinical score (SClinic) ranging from 0 to 15: Score 0 (mild): 0–2; Score 1 (moderate): 3–7; Score 2 (severe): 8–11; Score 3 (critical): 12–15 (Table 1).

Thoracic auscultation was performed using a stethoscope (3M Littmann Classic III, 3M Health Care, St. Paul, MN, USA). Both hemithoraces were systematically evaluated, with the left examined first, followed by the right. Cranial, medium (right side), and caudal lobes were assessed at predefined intercostal spaces. Findings were classified according to severity: Score 0: normal lung sounds; Score 1: bronchial breath sounds; Score 2: crepitations, wheezes, or rales; Score 3: absence of lung sounds. The final auscultatory score (SAusc) corresponded to the most severe abnormality detected (Table 1).

TUS was performed using an Esaote MyLab One Vet ultrasound system (Esaote España, Barcelona, Spain) equipped with a microconvex SC3123 probe. Reference frequency was set at 10 MHz with an imaging depth of 8 cm. Hydroalcoholic gel was applied to optimize acoustic coupling. Examinations were conducted in a dimly lit environment with lambs positioned in lateral recumbency. Both hemithoraces were evaluated using the same protocol, with the left examined first, followed by the right. Cranial, medium (right), and caudal lobes were examined using defined intercostal spaces and anatomical landmarks as reference points.

**Figure 1:**
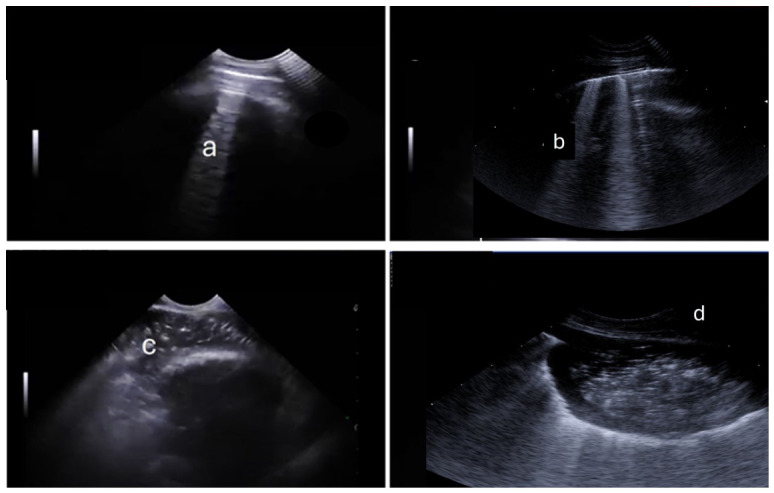
Representative thoracic ultrasonographic images illustrating each category of the SUlt scoring system in lambs. (**a**) Score 0 (normal): hyperechoic pleural line with A-line reverberation artifacts, indicating normal lung parenchyma; (**b**) Score 1 (mild): multiple B-lines (comet-tail artifacts) radiating from the pleural surface without consolidation, indicating interstitial syndrome; (**c**) Score 2 (moderate): focal pulmonary consolidation with hepatisation pattern; (**d**) Score 3 (severe): pulmonary abscess appearing as a hypoechoic encapsulated mass. Images reproduced from Sánchez-Fernández et al. [7] with permission.

Ultrasonographic findings were classified as: A-lines (normal), B-lines (comet-tail artifacts), consolidation (CON), pleural effusion (PE), and abscess (ABS) (Figure 1). The ultrasound score (SUlt) was assigned as follows: Score 0: normal lungs (A-lines only); Score 1: >5 sites with B-lines without consolidation; Score 2: >5 sites with B-lines and <5 sites with consolidation; Score 3: >5 sites with consolidation and/or pleural effusion or abscess (Table 1).

Gross pathological examination was performed at the abattoir 12 h after clinical, auscultatory, and ultrasonographic assessments. Pulmonary lesions were photographed and macroscopically evaluated according to distribution, margins, shape, color, size, texture, consistency, and extent. Lesion areas were delineated and quantified using Adobe^®^ Photoshop^®^ CC2023 (v24.0; Adobe Inc., San Jose, CA, USA). Pneumonia severity was classified according to percentage of lung consolidation (SPost): 0: <10%; 1: 10–20%; 2: 20–30%; 3: >30% (Table 1).

### 2.3. Bacteriological Analysis

Lung samples were collected post-mortem immediately after slaughter. Macroscopic lesions were recorded, and representative tissue samples were aseptically obtained from affected areas of the lung. Samples were individually labeled, transported to the laboratory under refrigerated conditions (4 °C), and processed within 24 h to preserve bacterial viability and minimize post-mortem overgrowth.

Bacteriological analyses were performed following standardized protocols for the isolation of respiratory pathogens. Each lung sample was processed individually to ensure traceability and avoid cross-contamination. The external surface of each specimen was aseptically decontaminated by searing with a sterile heated spatula. The tissue was then incised, and internal samples were collected using sterile swabs pre-moistened with 0.9% sterile saline. Samples were inoculated onto Columbia agar supplemented with 5% sheep blood (bioMérieux, Marcy-l’Étoile, France) and MacConkey agar (Oxoid, Basingstoke, UK) and incubated at 37 °C under microaerophilic conditions (5% O_2_, 10% CO_2_, 85% N_2_) for up to 48 h. Plates were examined at 24 and 48 h.

Bacterial identification was based on Gram staining, colony morphology, and hemolysis patterns, followed by biochemical characterization using the API20E system (bioMérieux, Marcy-l’Étoile, France) for Gram-negative isolates. Species-level identification was confirmed by MALDI-TOF MS (Bruker Daltonik, Bremen, Germany), according to the manufacturer’s instructions. To estimate relative bacterial abundance, growth was assessed semiquantitatively using the quadrant streak method and classified as sparse (first quadrant only), moderate (second quadrant), or heavy (third and fourth quadrants), as previously described [23].

The presence of *Mycoplasma ovipneumoniae* was assessed by quantitative polymerase chain reaction (qPCR). Genomic DNA was extracted using the E.Z.N.A.^®^ Tissue DNA Kit (Omega Bio-tek, Norcroos, GA, USA), following the manufacturer’s instructions with minor modifications. DNA extracted from ten subsamples per animal was pooled before analysis.

Quantitative PCR was performed on a QuantStudio™ 5 Real-Time PCR System (Thermo Fisher Scientific, Madrid, Spain) using the MycOvi qPCR (GPS™) kit (Genetic PCR Solutions™, Orihuela, Spain). The freeze-dried specific mixture was reconstituted with 105 µL resuspension buffer. The final amplification mixture (20 µL total volume) consisted of 9 µL DNase/RNase-free water, 5 µL GPS™-mix 4X, 1 µL specific qPCR mixture, and 5 µL template DNA. The amplification protocol comprised an initial activation step at 95 °C for 2 min, followed by 40 cycles of denaturation at 95 °C for 5 s and hybridization/extension at 60 °C for 20 s. A no-template control (NTC) and a positive control were included in each run.

Ct values were used for both descriptive characterisation of *Mycoplasma ovipneumoniae* DNA load and as an ordinal predictor in supplementary multivariable models (see Section 2.4 and Section 3.5). Ct values are assay-dependent and should be interpreted alongside clinical findings and standardised reference curves.

### 2.4. Statistical Analysis

Statistical analyses were performed using IBM SPSS Statistics (version 31.0.1.0; IBM Corp., Armonk, NY, USA). Two-sided *p*-values < 0.05 were considered statistically significant, and all 80 animals had complete data for all study variables (severity scores, pathogen detection, and coinfection status) and were included in the analysis. Disease severity was assessed using four complementary 0–3 ordinal scoring systems: clinical severity (SClinic), auscultatory severity (SAusc), ultrasonographic severity (SUlt), and post-mortem severity (SPost), with higher categories reflecting more severe pulmonary involvement. Data distribution was evaluated using the Kolmogorov–Smirnov test. Continuous variables are reported as mean ± standard deviation (SD) or median and interquartile range (IQR), as appropriate. Categorical variables are summarised as absolute frequencies and percentages with 95% confidence intervals (95% CIs) for prevalence estimates, calculated by the Wilson method. Microorganisms identified in fewer than 10% of animals were grouped as “other detected microorganisms” for descriptive purposes and were not entered individually into multivariable models.

Because each severity outcome was ordinal, four separate multivariable proportional odds (cumulative logit) models were fitted with SClinic, SAusc, SUlt, and SPost as the dependent variable. All models included the same set of a priori–defined predictors: detection of *Mycoplasma ovipneumoniae*, *Pasteurella multocida*, *Mannheimia haemolytica*, and *Trueperella pyogenes*, together with coinfection status (≥2 pathogens detected simultaneously in the same animal). Because coinfection status was derived from the detection of individual pathogens, some conceptual overlap between predictors may exist. Nevertheless, coinfection status was retained to evaluate the overall burden of pathogen co-occurrence while simultaneously estimating the independent association of each pathogen with disease severity. To reduce sparse-cell bias and to avoid unstable estimates, no further variable-selection procedures (forward, backward, or stepwise) were applied. Variance inflation factors (VIF) were computed for all predictors; VIF < 3 indicated no meaningful multicollinearity, supporting the inclusion of all predictors simultaneously.

The proportional odds assumption was tested for each model using the Test of Parallel Lines in the SPSS PLUM procedure, supplemented by visual inspection of the empirical logits. Model fit was evaluated with the Pearson chi-square goodness-of-fit test, the deviance test, and the Nagelkerke pseudo-R^2^. Influential observations were screened using standardised residuals and Cook’s distance. No major violations of model assumptions were identified. Exact regression methods were considered during model development. However, model convergence was satisfactory, sparse-cell problems were not observed, and standard maximum-likelihood estimation was therefore retained for all analyses. Adjusted odds ratios (aORs) with 95% CIs were derived from each model; values > 1 indicate increased odds of being in a higher severity category. Because four correlated outcomes were analysed, Benjamini–Hochberg false discovery rate (FDR) correction [25] was applied across the four primary models; both raw and FDR-adjusted *p*-values are reported.

Given the moderate sample size and the low prevalence of some microorganisms, emphasis was placed on effect sizes and 95% confidence intervals rather than on *p*-values alone. To minimise the risk of overfitting, the number of predictors included in the multivariable models was restricted a priori. Model stability was assessed through evaluation of model convergence, confidence intervals, residual diagnostics, and goodness-of-fit statistics. Results are reported following the STROBE-Vet guidelines. Adjusted odds ratios and corresponding 95% CIs are graphically summarised as forest plots, with the reference line at aOR = 1.

In a supplementary analysis, Ct values were categorised into four ordinal groups (Ct 0, Ct 1, Ct 2, Ct 3) reflecting increasing *Mycoplasma ovipneumoniae* DNA load, and entered as an ordinal predictor in four additional multivariable proportional odds models. In these models, the binary *Mycoplasma ovipneumoniae* variable was excluded to avoid collinearity with SPCR Ct ≥ 1. FDR correction was applied across the four outcomes. Spearman’s rank correlation and Kruskal–Wallis tests were used to assess the monotonic association between SPCR Ct category and severity scores. A two-sided *p*-value < 0.05 was considered statistically significant throughout.

## 3. Results

### 3.1. Study Population and Distribution of Disease Severity

A total of 80 lambs were included in the study. The distribution of clinical, auscultatory, ultrasonographic, and post-mortem severity scores is presented in Table 2. Clinical and auscultatory evaluations were predominantly classified as mild to moderate disease (Scores 1 and 2), whereas ultrasonographic and post-mortem examinations identified a higher proportion of severe lesions. Ultrasonography classified 31.3% of animals as Score 3, while severe post-mortem lesions were observed in 27.5% of the study population (Table 2).

### 3.2. Prevalence of Respiratory Pathogens and Coinfections

**Figure 2:**
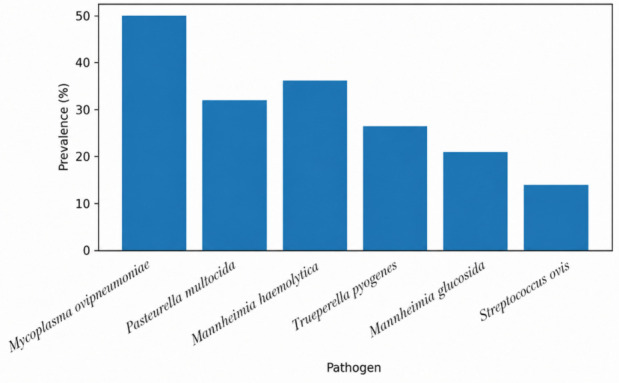
Prevalence of respiratory pathogens detected in lung samples. Bars represent the percentage of animals positive for each bacterial species identified in the study population.

The frequency of microorganisms detected in lung samples is summarised in Table 3 and illustrated in Figure 2. *Mycoplasma ovipneumoniae* was the most frequently detected pathogen, being identified in 41 of 80 animals (51.3%). Other commonly detected microorganisms included *Mannheimia haemolytica* (35.4%), *Pasteurella multocida* (31.6%), and *Trueperella pyogenes* (29.1%). *Mannheimia glucosida* and *Streptococcus ovis* were detected less frequently, whereas additional bacterial species were grouped as “other detected microorganisms” because of their low prevalence.

Coinfections were common, occurring in 48 animals (60.8%). As shown in Table 4, detection of multiple pathogens was frequent, with 30% of animals harbouring two pathogens and a further 30% harbouring three or more pathogens. Conversely, 39.2% of animals harboured a single bacterial species (17.7%) or no detectable bacterial pathogen (21.5%), and were therefore classified as non-coinfected for the purposes of this analysis. These findings support the polymicrobial nature of ovine respiratory disease in the study population.

### 3.3. Distribution of Severity Scores According to Pathogen Detection

**Figure 3:**
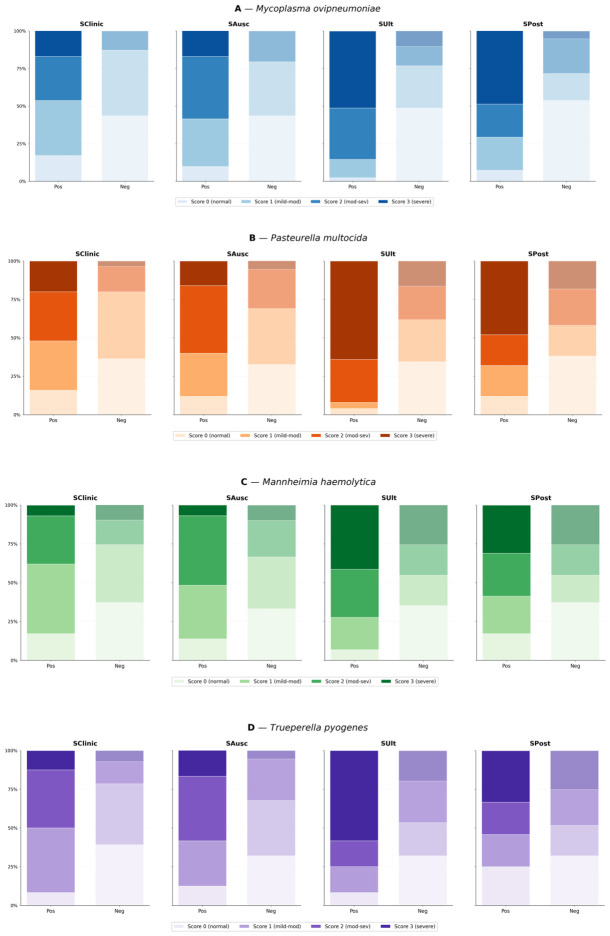

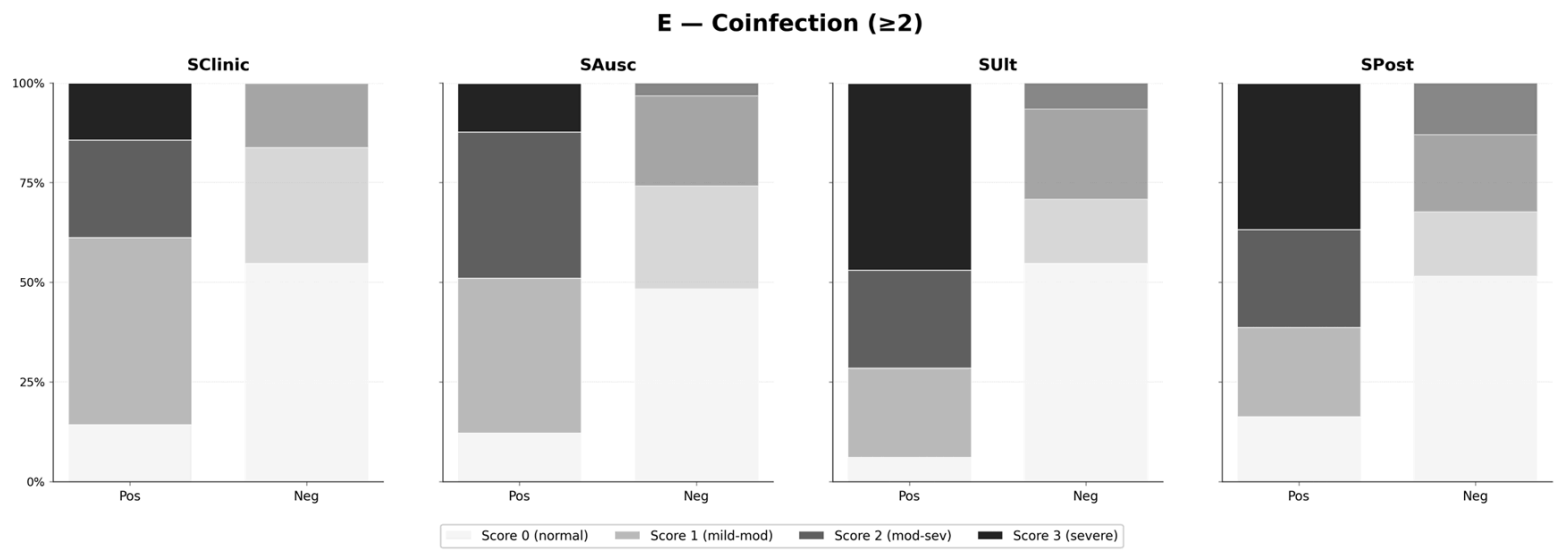
Distribution of disease severity scores according to microorganism detection. (**A**): *Mycoplasma ovipneumoniae*; (**B**): *Pasteurella multocida*; (**C**): *Mannheimia haemolytica*; (**D**): *Trueperella pyogenes* and (**E**): Coinfection. Stacked bars show the percentage of animals in each severity category (Scores 0–3) among microorganism-negative and positive animals. Panels represent clinical, auscultatory, ultrasonographic, and post-mortem severity scores.

The distribution of clinical, auscultatory, ultrasonographic, and post-mortem severity scores (SClinic, SAusc, SUlt, and SPost, respectively) stratified by pathogen detection is shown in Figure 3 (panels A–E). The proportions of animals in each severity category (Score 0, normal; Score 1, mild-moderate; Score 2, moderate-severe; Score 3, severe) are displayed for pathogen-positive and pathogen-negative groups.

Across all five pathogen/condition panels, the largest differences between positive and negative animals were consistently observed for ultrasonographic assessment (SUlt), followed by post-mortem evaluation (SPost). *Pasteurella multocida* and *Mycoplasma ovipneumoniae* showed the strongest shifts toward severe (Score 3) ultrasonographic and post-mortem lesions, whereas *Mannheimia haemolytica* and *Trueperella pyogenes* produced more moderate gradients. Coinfection was associated with an overall increase in severity across all four scoring systems. These descriptive patterns are consistent with the multivariable ordinal logistic regression results presented in Section 3.4.

#### 3.3.1. *Mycoplasma ovipneumoniae* (Figure 3A)

Among the 41 animals positive for *Mycoplasma ovipneumoniae*, the proportion in the two highest severity categories (Scores 2–3) increased progressively from clinical to post-mortem assessment. SClinic Scores 2–3 were recorded in 46.4% of positive animals compared with 32.7% of negative animals. SAusc Scores 2–3 were present in 58.5% of positive animals versus 41.8% of negative animals. The most pronounced shift was observed in SUlt, where 51.2% of positive animals were classified as Score 3 and 85.4% as Scores 2–3, whereas only 10.3% and 51.3% of negative animals fell into these categories, respectively. A comparable pattern was observed for SPost, with 48.8% of positive animals showing severe (Score 3) lesions compared with 5.1% of negative animals, and 70.7% in Scores 2–3 versus 46.1% in negative animals.

#### 3.3.2. *Pasteurella multocida* (Figure 3B)

The 27 positive animals for *Pasteurella multocida* exhibited the highest ultrasonographic severity among all evaluated pathogens. SUlt Score 3 was observed in 64.0% of positive animals, with a combined Score 2–3 proportion of 92.0%, compared with 16.4% and 48.0% in negative animals, respectively. For SPost, 48.0% of positive animals presented with severe lesions (Score 3) and 68.0% with Scores 2–3, versus 18.2% and 56.4% in negative animals. The SClinic and SAusc distributions showed milder but consistent trends, with 52.0% and 60.0% of positive animals in Scores 2–3, respectively.

#### 3.3.3. *Mannheimia haemolytica* (Figure 3C)

Among the 29 animals positive for *Mannheimia haemolytica*, the severity gradient was less pronounced for clinical and auscultatory scores, with 37.9% and 51.7% of positive animals in Scores 2–3 for SClinic and SAusc, respectively. The differences became evident in SUlt, where 41.4% of positive animals were classified as Score 3 and 72.4% as Scores 2–3, compared with 25.5% and 51.0% in negative animals. SPost severity was similar between positive and negative groups (31.0% vs. 25.5% in Score 3; 58.7% vs. 62.7% in Scores 2–3), suggesting a weaker association with macroscopic lung lesions.

#### 3.3.4. *Trueperella pyogenes* (Figure 3D)

The 24 animals positive for *Trueperella pyogenes* showed a distinct pattern in which ultrasonographic severity was markedly elevated, with 58.3% of positive animals in Score 3 and 75.0% in Scores 2–3, compared with 19.6% and 51.1% in negative animals, respectively. SPost distribution indicated 33.3% of positive animals with Score 3 lesions and 54.2% with Scores 2–3, compared with 25.0% and 50.0% in negative animals. SClinic and SAusc distributions also showed moderate shifts toward higher categories, with 50.0% and 58.3% of positive animals in Scores 2–3, respectively.

#### 3.3.5. Coinfection (Figure 3E)

The 49 animals with detection of two or more pathogens (Coinfection ≥ 2) showed an intermediate but consistent trend toward higher severity across all four scoring systems. SClinic Scores 2–3 were recorded in 38.8% of coinfected animals versus 27.4% of non-coinfected animals, and SAusc Scores 2–3 in 48.9% versus 31.2%. SUlt Score 3 lesions were present in 46.9% of coinfected animals and 71.5% in Scores 2–3, compared with 6.5% and 25.8% in non-coinfected animals, respectively. SPost severity was also higher, with 36.7% of coinfected animals in Score 3 and 61.3% in Scores 2–3, versus 12.9% and 45.2% in non-coinfected animals.

The biggest differences were observed for ultrasonographic and post-mortem evaluations. Among animals positive for *Mycoplasma ovipneumoniae*, 51.2% were classified as Score 3 by ultrasonography and 48.8% as Score 3 during post-mortem examination. In comparison, severe lesions represented 31.6% and 27.8% of the overall study population for ultrasonographic and post-mortem scores, respectively (Table 2).

A similar trend toward increasing disease severity was observed among animals positive for *Pasteurella multocida*, *Trueperella pyogenes*, and *Mannheimia haemolytica*. Severe ultrasonographic lesions (Score 3) were identified in 64.0% of animals positive for *Pasteurella multocida* and 60.9% of animals positive for *Trueperella pyogenes*. Likewise, severe post-mortem lesions were observed in 48.0% and 34.8% of positive animals, respectively.

Coinfected animals also showed a tendency toward greater disease severity. Severe ultrasonographic lesions were present in 47.9% of coinfected animals, while 37.5% exhibited severe post-mortem lesions, further supporting the contribution of pathogen co-occurrence to disease expression.

Overall, these descriptive findings suggest possible associations between pathogen detection and increasing respiratory disease severity, particularly for *Mycoplasma ovipneumoniae*, although formal multivariable modelling is required to evaluate these relationships.

### 3.4. Multivariable Ordinal Logistic Regression Analysis

**Figure 4:**
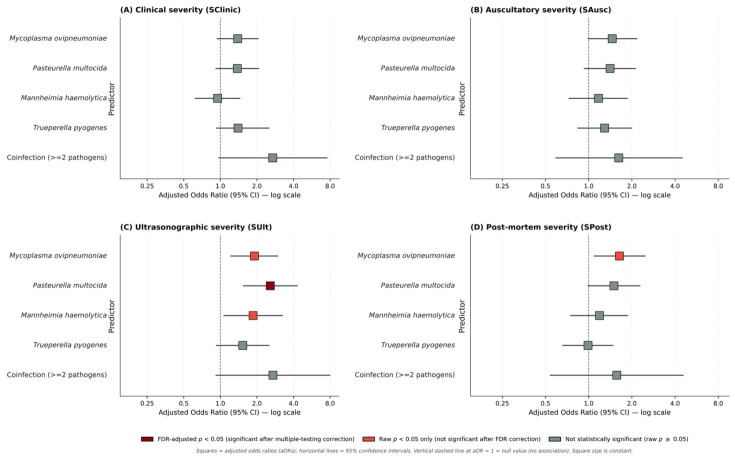
Effect sizes from multivariable ordinal logistic regression models. Forest plots showing adjusted odds ratios (aORs) and 95% confidence intervals (95% CIs) obtained from multivariable proportional odds ordinal logistic regression models for (**A**) clinical severity score (SClinic), (**B**) auscultatory severity score (SAusc), (**C**) ultrasonographic severity score (SUlt), and (**D**) post-mortem severity score (SPost). Odds ratios greater than 1 indicate increased odds of belonging to a higher disease severity category (reference category: severity score = 0; pathogen not detected). The vertical dashed line represents an odds ratio of 1. Footnote: aOR, adjusted odds ratio; CI, confidence interval. All models included *Mycoplasma ovipneumoniae*, *Pasteurella multocida*, *Mannheimia haemolytica*, *Trueperella pyogenes*, and coinfection status simultaneously. Odds ratios and 95% confidence intervals are displayed for each predictor.

The results of the multivariable ordinal logistic regression models are presented in Table 5, Table 6, Table 7 and Table 8 and summarised in Figure 4.

For the clinical severity score (SClinic), none of the evaluated pathogens showed a statistically significant independent association with increasing disease severity after multivariable adjustment. The closest trend was observed for coinfection status (aOR = 2.70; 95% CI: 0.97–7.56; raw *p* = 0.070; FDR-adjusted *p* = 0.166), followed by *Mycoplasma ovipneumoniae* (aOR = 1.39; 95% CI: 0.94–2.05; raw *p* = 0.101), *Pasteurella multocida* (aOR = 1.38; 95% CI: 0.92–2.07; raw *p* = 0.119), *Trueperella pyogenes* (aOR = 1.40; 95% CI: 0.93–2.52; raw *p* = 0.186), and *Mannheimia haemolytica* (aOR = 0.95; 95% CI: 0.62–1.45; raw *p* = 0.813) (Table 5).

Similarly, for the auscultatory severity score (SAusc), no pathogen remained significantly associated with increasing severity after multivariable adjustment. *Mycoplasma ovipneumoniae* showed a borderline association (aOR = 1.46; 95% CI: 0.99–2.17; raw *p* = 0.058), while the remaining predictors showed no evidence of association (all raw *p* > 0.10). Coinfection was not associated with auscultatory severity (aOR = 1.62; 95% CI: 0.59–4.50; raw *p* = 0.350) (Table 6).

The strongest associations were observed for ultrasonographic severity (SUlt). At the raw *p* < 0.05 level, three pathogens showed significant associations with higher ultrasonographic severity: *Pasteurella multocida* (aOR = 2.58; 95% CI: 1.55–4.31; raw *p* < 0.001), *Mycoplasma ovipneumoniae* (aOR = 1.91; 95% CI: 1.22–2.97; raw *p* = 0.005), and *Mannheimia haemolytica* (aOR = 1.86; 95% CI: 1.07–3.25; raw *p* = 0.029). Coinfection also showed a positive trend (aOR = 2.71; 95% CI: 0.92–7.97; raw *p* = 0.070), whereas Trueperella pyogenes was not associated (aOR = 1.53; 95% CI: 0.93–2.52; raw *p* = 0.098) (Table 7).

For post-mortem lesion severity (SPost), only *Mycoplasma ovipneumoniae* showed a significant raw association (aOR = 1.64; 95% CI: 1.09–2.48; raw *p* = 0.017). *Pasteurella multocida* displayed a borderline association (aOR = 1.50; 95% CI: 0.99–2.28; raw *p* = 0.057), and the remaining pathogens showed no evidence of association (all raw *p* > 0.40). Coinfection was not significantly associated with post-mortem severity (aOR = 1.57; 95% CI: 0.54–4.56; raw *p* = 0.407) (Table 8).

After Benjamini–Hochberg false discovery rate (FDR) correction [25] across the 20 individual tests (5 predictors × 4 outcomes), only the association between *Pasteurella multocida* and ultrasonographic severity remained statistically significant (raw *p* < 0.001; FDR-adjusted *p* = 0.010). *Mycoplasma ovipneumoniae* in ultrasonographic severity was borderline (FDR-adjusted *p* = 0.050). All other reported associations should therefore be interpreted as exploratory and require confirmation in independent studies with larger sample sizes.

### 3.5. Association Between Mycoplasma ovipneumoniae Bacterial Load (SPCR Ct) and Disease Severity

The semiquantitative assessment of *Mycoplasma ovipneumoniae* DNA load by quantitative PCR was performed on all 80 animals. Cycle threshold (Ct) values were categorised into four ordinal groups: Ct 0 (Ct > 40 or undetectable, *n* = 36, 45.0%), Ct 1 (high Ct/low bacterial load, *n* = 4, 5.0%), Ct 2 (intermediate Ct, *n* = 12, 15.0%), and Ct 3 (low Ct/high bacterial load, *n* = 28, 35.0%). All 44 animals with Ct ≥ 1 were confirmed as *Mycoplasma ovipneumoniae*-positive by bacteriological methods, indicating that the SPCR Ct score provides a finer-grained quantification of bacterial load than the binary classification alone.

#### 3.5.1. Descriptive Distribution

**Figure 5:**
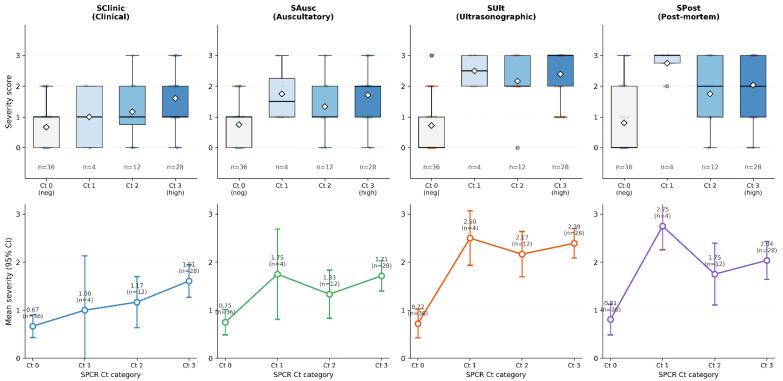
Severity scores across SPCR Ct categories for *Mycoplasma ovipneumoniae* (Top: boxplots with individual data points; Bottom: mean 95% Ct trend lines).

The cross-tabulation of SPCR Ct categories against the four severity outcomes is shown in Table 9 and visualised in Figure 5. A clear dose–response relationship was observed: the proportion of animals classified as Score 3 (severe) increased progressively from Ct 0 to Ct 3 for all four outcomes. For ultrasonographic severity (SUlt), the proportion of severe lesions (S3) rose from 5.6% in Ct 0 to 60.7% in Ct 3. For post-mortem severity (SPost), the corresponding values were 5.6% and 46.4%. Mean severity scores also increased monotonically with Ct category (Figure 5, lower panels): SClinic rose from 0.67 (Ct 0) to 1.61 (Ct 3); SUlt rose from 0.72 to 2.39; SPost rose from 0.81 to 2.04.

#### 3.5.2. Trend Tests

The Spearman rank correlation between SPCR Ct (as ordinal 0–3) and severity score was positive and statistically significant for all four outcomes: SClinic ρ = +0.443 (*p* < 0.001), SAusc ρ = +0.454 (*p* < 0.001), SUlt ρ = +0.652 (*p* < 0.001), and SPost ρ = +0.471 (*p* < 0.001). Kruskal–Wallis non-parametric tests confirmed the heterogeneity of severity distributions across SPCR Ct categories for all four outcomes (all *p* ≤ 0.0014), with the strongest discrimination observed for SUlt (H = 37.5, *p* < 0.0001).

#### 3.5.3. Multivariable Ordinal Logistic Regression

To assess the independent effect of *Mycoplasma ovipneumoniae* bacterial load on disease severity, four separate multivariable proportional odds (cumulative logit) models were fitted. Each model included SPCR Ct (ordinal 0–3) as the main predictor, adjusted for *Pasteurella multocida*, *Mannheimia haemolytica*, *Trueperella pyogenes*, and coinfection status (≥2 pathogens). Because SPCR Ct ≥ 1 is essentially equivalent to *Mycoplasma ovipneumoniae* detection, the binary *Mycoplasma ovipneumoniae* variable was excluded to avoid collinearity.

The results (Table 10) show that SPCR Ct was an independent predictor of severity for all four outcomes after adjustment for other pathogens. The adjusted odds ratio (aOR) for a one-unit increase in SPCR Ct category was 1.43 (95% CI: 0.97–2.10; *p* = 0.070) for SClinic, 1.55 (95% CI: 1.05–2.28; *p* = 0.027) for SAusc, 1.98 (95% CI: 1.30–3.01; *p* = 0.0015) for SUlt, and 1.73 (95% CI: 1.16–2.57; *p* = 0.007) for SPost. The strongest association was observed for ultrasonographic severity, where each one-unit increase in SPCR Ct approximately doubled the odds of being in a higher severity category.

#### 3.5.4. Comparison with Binary Classification

When SPCR Ct was entered as an ordinal predictor, the resulting aORs were larger and more statistically robust than those obtained with the binary *Mycoplasma ovipneumoniae* classification in the previous section (Section 3.4, Table 5, Table 6, Table 7 and Table 8). For SUlt, the binary *Mycoplasma ovipneumoniae* aOR was 1.91 (95% CI: 1.22–2.97; *p* = 0.005), whereas the ordinal SPCR Ct yielded aOR = 1.98 (95% CI: 1.30–3.01; *p* = 0.0015) per unit increase. This indicates that SPCR Ct captures additional dose-related information not provided by the binary classification.

SPCR Ct is strongly and independently associated with disease severity across all four scoring systems, with the strongest effect on ultrasonographic findings. The dose–response pattern, supported by both non-parametric trend tests and multivariable ordinal regression, suggests that *Mycoplasma ovipneumoniae* bacterial load, rather than mere presence, is a key driver of disease severity in this cohort. These findings underscore the clinical value of quantitative PCR for risk stratification and disease monitoring in ORC.

## 4. Discussion

The present study investigated the association between respiratory pathogen detection and disease severity in lambs using clinical, auscultatory, ultrasonographic, and post-mortem scoring systems. By integrating microbiological findings with multiple complementary diagnostic approaches and applying multivariable ordinal logistic regression models, this study sought to evaluate the relationship between selected bacterial pathogens and respiratory disease severity within the context of the Ovine Respiratory Complex (ORC). Unlike previous investigations that primarily relied on descriptive or univariable analyses, the present work attempted to account for pathogen co-occurrence and the ordinal nature of disease severity, thereby providing a more comprehensive assessment of factors associated with respiratory lesions in naturally infected animals [3,16].

The most robust finding of the present study was the association between *Pasteurella multocida* detection and increasing ultrasonographic severity. Importantly, this was the only association that remained statistically significant after adjustment for multiple testing using the Benjamini–Hochberg false discovery rate procedure [25]. *Pasteurella multocida* is widely recognized as an important opportunistic respiratory pathogen in sheep and has frequently been isolated from lungs exhibiting pneumonic lesions [20,26,27,28]. Previous studies have demonstrated a strong association between *Pasteurella multocida* isolation and pulmonary consolidation in ovine pneumonic pasteurellosis [20]. The present findings extend this evidence by suggesting that *Pasteurella multocida* detection is associated with more severe pulmonary abnormalities identifiable through thoracic ultrasonography. Because ultrasonography allows for the detection of active pulmonary consolidation and pleural pathology, it may be particularly useful for identifying disease manifestations associated with this pathogen [7,8,9,10,11,12].

Several additional associations were identified in the multivariable models before correction for multiple testing. In particular, *Mycoplasma ovipneumoniae* detection was associated with increasing ultrasonographic and post-mortem severity, while *Mannheimia haemolytica* showed an association with ultrasonographic severity. However, none of these associations remained statistically significant after false discovery rate correction and should therefore be interpreted as exploratory findings. Although these trends are biologically plausible and consistent with current understanding of ovine respiratory disease pathogenesis, the present study does not provide sufficient statistical evidence to confirm independent associations after accounting for multiple comparisons. Consequently, these observations should be regarded as hypothesis-generating and warrant confirmation in larger studies [29,30,31].

To further explore whether the *Mycoplasma ovipneumoniae* severity association reflected a dose–response relationship rather than a simple presence/absence effect, a supplementary analysis was conducted using SPCR Ct (an ordinal 0–3 measure of bacterial DNA load) as the main predictor (Section 3.5). After adjustment for *Pasteurella multocida*, *Mannheimia haemolytica*, *Trueperella pyogenes*, and coinfection status, each one-unit increase in SPCR Ct category was associated with significantly higher odds of increased ultrasonographic severity (aOR = 1.98; 95% CI: 1.30–3.01; *p* = 0.0015) and post-mortem severity (aOR = 1.73; 95% CI: 1.16–2.57; *p* = 0.007). The dose–response pattern was confirmed by a strong Spearman correlation with ultrasonographic severity (ρ = +0.652) and by a significant Kruskal–Wallis test (H = 37.5; *p* < 0.0001). Notably, the ordinal SPCR Ct model yielded larger effect sizes and more robust *p*-values than the binary *Mycoplasma ovipneumoniae* model for the same outcomes, indicating that quantitative PCR provides additional prognostic information beyond simple detection. These findings are consistent with the known biology of *Mycoplasma ovipneumoniae*, in which higher bacterial loads in the lower respiratory tract are expected to produce more extensive epithelial damage, greater impairment of mucociliary clearance, and consequently more severe ultrasonographic and post-mortem lesions. From a clinical perspective, SPCR Ct may therefore serve as a useful biomarker for risk stratification and longitudinal disease monitoring in sheep flocks affected by ORC, and may inform future antimicrobial stewardship strategies pending susceptibility testing and longitudinal outcome data.

The exploratory associations observed for *Mycoplasma ovipneumoniae* are nevertheless noteworthy because this microorganism was the most frequently detected pathogen in the study population. *Mycoplasma ovipneumoniae* has long been considered a key component of respiratory disease complexes in domestic sheep and wild *Caprinae* species [1,29,30,31,32]. Experimental and epidemiological studies have demonstrated that this pathogen impairs mucociliary clearance, modulates pulmonary immune responses, and facilitates colonization by secondary bacterial pathogens [30,33,34]. Furthermore, *Mycoplasma ovipneumoniae* has been associated with chronic respiratory disease, reduced growth performance, and increased susceptibility to polymicrobial pneumonia [1,29,31,35]. Therefore, although the present results cannot establish an independent association after multiple-testing correction, the observed trends remain biologically credible and should be explored further in adequately powered investigations.

Similarly, the exploratory association observed for *Mannheimia haemolytica* is consistent with its established role as a major pathogen in ovine pneumonic pasteurellosis [15,17,18,19,36,37,38,39,40]. The pathogenicity of *Mannheimia haemolytica* is largely mediated by leukotoxin production and the induction of severe inflammatory responses within the lung parenchyma [18,19,40]. However, the absence of statistically significant findings after correction for multiple testing suggests that its independent contribution to lesion severity could not be conclusively demonstrated in the present dataset.

*Trueperella pyogenes* was detected in 28.8% of animals and showed a notable association with ultrasonographic severity, which is consistent with its recognised role as an opportunistic pathogen in the respiratory tract of sheep and other livestock [41,42,43]. The pathogenic potential of *T. pyogenes* is primarily mediated by pyolysin, a pore-forming toxin that induces cell death in a wide range of host cells, as well as by neuraminidases, fimbriae, and biofilm formation capacity that facilitate tissue invasion and colonisation [41,43]. The predominance of heavy bacterial growth in *T. pyogenes*-positive samples suggests that high bacterial loads may be necessary for significant pulmonary involvement.

An important observation emerging from this study is that associations between pathogen detection and disease severity were most evident when severity was assessed by thoracic ultrasonography. Ultrasonographic outcomes produced the largest effect sizes and the greatest number of statistically significant or near-significant associations among the evaluated diagnostic methods. These findings are consistent with previous reports demonstrating the superior sensitivity of thoracic ultrasonography for detecting pulmonary consolidation and pleural abnormalities compared with conventional clinical examination and auscultation [7,8,9,10,11,12,44,45,46]. Furthermore, the close agreement observed between ultrasonographic and post-mortem findings supports the growing evidence that thoracic ultrasonography is a reliable, non-invasive method for assessing pulmonary lesions under field conditions [7,12,44,45,46].

Coinfections were identified in approximately 60% of animals, confirming the polymicrobial nature of ORC. Similar observations have been reported previously, where respiratory disease results from complex interactions among bacterial pathogens, host immunity, management practices, environmental stressors, and, potentially, viral infections [3,4,6,16,47,48]. Animals harboring multiple pathogens tended to present more severe lesions in descriptive analyses. Nevertheless, coinfection status was not independently associated with increasing severity after multivariable adjustment. This finding suggests that disease severity may depend not only on the number of pathogens present but also on the biological characteristics of individual microorganisms and the nature of pathogen–pathogen interactions. Because coinfection status was derived from the same pathogen detections included as individual predictors, some degree of conceptual overlap between variables should be acknowledged. Consequently, the estimated effects of coinfection should be interpreted cautiously and primarily as an indicator of the overall burden of pathogen co-occurrence rather than as an entirely independent biological factor.

The absence of significant associations for clinical and auscultatory severity after multivariable adjustment further highlights the limited specificity of these diagnostic approaches for identifying etiological agents. Clinical signs and auscultatory abnormalities are influenced by numerous factors, including disease stage, lesion chronicity, environmental conditions, and host immune responses [7,12]. Consequently, these methods may be less capable of discriminating infections caused by different respiratory pathogens than imaging-based assessments.

Several limitations should be considered when interpreting the present findings. First, the relatively small sample size may have limited statistical power to detect modest associations and likely contributed to the attenuation of significance after correction for multiple testing. Although the number of predictors included in the multivariable models was restricted a priori to minimise the risk of overfitting, some associations may have remained undetected because of limited statistical power. Second, the cross-sectional design precludes causal inference and allows only the identification of statistical associations between pathogen detection and disease severity. Accordingly, pathogen detection should not be interpreted as evidence of causation but rather as evidence of statistical association with disease severity at the time of evaluation. Longitudinal studies will be required to determine the temporal and causal relationships between pathogen presence and disease progression. Third, microbiological detection cannot differentiate active pathogenic involvement from asymptomatic carriage or secondary colonization, particularly for opportunistic organisms commonly present within the respiratory microbiota [22]. Fourth, viral pathogens, which are recognized contributors to respiratory disease complexes in small ruminants, were not evaluated and may represent important unmeasured confounding factors [3,4]. Finally, the study population consisted of convenience-selected animals with clinical or productive abnormalities and, therefore, should not be considered representative of the wider population of fattening lambs.

Despite these limitations, the present study contributes valuable information regarding the relationship between respiratory pathogen detection and multiple indicators of disease severity in naturally affected lambs. By integrating microbiological analyses with clinical, ultrasonographic, and pathological evaluations, the study provides further support for the use of thoracic ultrasonography as a sensitive diagnostic tool and highlights the complexity of pathogen interactions within ORC. Future investigations incorporating larger sample sizes, longitudinal follow-up, quantitative pathogen load assessment, and viral diagnostics will be necessary to clarify the independent contribution of individual pathogens to respiratory disease severity in sheep. These findings were obtained from a convenience-selected cohort of clinically abnormal fattening lambs and should not be extrapolated to the general fattening lamb population. Future studies including randomly selected animals are needed to establish population-level prevalence estimates.

## 5. Conclusions

Respiratory disease in lambs was characterised by frequent pathogen co-occurrence, supporting the polymicrobial nature of the syndrome. Among the pathogens evaluated, Pasteurella multocida showed the most robust independent association with disease severity, specifically with increasing ultrasonographic severity, and was the only association to remain statistically significant after Benjamini–Hochberg false discovery rate correction. Mycoplasma ovipneumoniae bacterial load (SPCR Ct) was independently associated with higher ultrasonographic and post-mortem severity in a dose–response pattern, supporting the clinical value of quantitative PCR for risk stratification. Thoracic ultrasonography proved to be a valuable tool for detecting severe pulmonary lesions and showed close agreement with post-mortem findings.

## Figures and Tables

**Table 1:** Summary of scoring systems used in the study.

Score	Parameters Evaluated	Score 0	Score 1	Score 2	Score 3
Clinical Score (SClinic)	Ocular discharge, Nasal discharge, Head tilting, Cough, Rectal temperature	0–normal	1–4 (mild/moderate)	5–10 (moderate/severe)	11–15 (severe)
Auscultatory Score (SAusc)	Lung sounds on thoracic auscultation	Normal lung sounds	Bronchial breath sounds	Crepitations, wheezes, or rales	Absence of lung sounds
Ultrasound Score (SUlt)	A-lines ^1^, B-lines ^2^, Consolidation, Pleural effusion, Abscess	Normal lungs	>5 sites with B-lines, no consolidation	>5 sites with B-lines + <5 sites with consolidation	>5 sites with consolidation and/or pleural effusion or abscess
Post-mortem Score (SPost)	Percentage of lung consolidation	<10%	10–20%	20–30%	>30%

^1^ A-lines: normal lungs, ^2^ B-lines: comet lines.

**Table 2:** Distribution of clinical, auscultatory, ultrasonographic, and post-mortem severity scores in the study population (*n* = 80).

Severity Score	SClinic *n* (%)	SAusc *n* (%)	SUlt *n* (%)	SPost *n* (%)
0 (normal)	19 (23.8)	15 (18.8)	17 (21.3)	25 (31.3)
1 (mild-moderate)	27 (33.8)	24 (30.0)	15 (18.8)	18 (22.5)
2 (moderate-severe)	23 (28.7)	27 (33.8)	23 (28.7)	15 (18.8)
3 (severe)	11 (13.8)	14 (17.5)	25 (31.3)	22 (27.5)
Total	80 (100.0)	80 (100.0)	80 (100.0)	80 (100.0)

SClinic = clinical respiratory severity score; SAusc = auscultatory severity score; SUlt = ultrasonographic severity score; SPost = post-mortem severity score. Scores ranged from 0 to 3, with increasing values indicating greater disease severity. Data are presented as the number of animals (*n*) and percentage (%).

**Table 3:** Frequency of microorganisms detected in lung samples and coinfection status (*n* = 80).

Microorganism	*n* Positive	%	95% CI (Wilson)
*Mycoplasma ovipneumoniae*	41	51.3	40.4–62.0
*Mannheimia haemolytica*	28	35.0	25.3–46.0
*Pasteurella multocida*	25	31.3	22.0–42.2
*Trueperella pyogenes*	23	28.8	19.9–39.6
*Mannheimia glucosida*	8	10.0	5.2–18.5
*Streptococcus ovis*	5	6.3	2.7–13.7
Other detected microorganisms *	12	15.0	8.8–24.4
Coinfection (≥2 pathogens)	48	60.0	49.0–70.1

Prevalence was calculated using the total study population (*n* = 80). Coinfection was defined as the simultaneous detection of two or more respiratory pathogens in the same animal. Other detected microorganisms included bacterial species identified at low frequency and therefore not analysed individually in multivariable models. * Other detected microorganisms included bacterial species detected at low frequency, including *Bibersteinia trehalosi*, *Escherichia coli*, *Bordetella bronchiseptica*, *Corynebacterium* spp., *Pasteurella stomatis*, *Actinobacillus* spp., and other isolates that were not analysed individually because of their low prevalence.

**Table 4:** Number of respiratory pathogens detected per animal (*n* = 80).

Number of Pathogens	Animals (*n*)	Percentage (%)
1 pathogen	32	40.0
2 pathogens	24	30.0
≥3 pathogens	24	30.0
Total	80	100.0

Coinfection was defined as the simultaneous detection of two or more respiratory pathogens in the same animal. Percentages were calculated using the total study population (*n* = 80).

**Table 5:** Multivariable ordinal logistic regression analysis for clinical severity score (SClinic).

Predictor	aOR	95% CI	*p*-Value (Raw)	*p*-Value (FDR-Adjusted)
*Mycoplasma ovipneumoniae*	1.39	0.94–2.05	0.101	0.158
*Pasteurella multocida*	1.38	0.92–2.07	0.132	0.189
*Mannheimia haemolytica*	0.95	0.62–1.45	0.813	1.000
*Trueperella pyogenes*	1.40	0.93–2.52	0.098	0.158
Coinfection (≥2 pathogens)	2.70	0.97–7.56	0.070	0.156

**Table 6:** Multivariable ordinal logistic regression analysis for auscultatory severity score (SAusc).

Predictor	aOR	95% CI	*p*-Value (Raw)	*p*-Value (FDR-Adjusted)
*Mycoplasma ovipneumoniae*	1.46	0.99–2.17	0.058	0.156
*Pasteurella multocida*	1.41	0.93–2.12	0.103	0.158
*Mannheimia haemolytica*	1.17	0.73–1.86	0.524	0.552
*Trueperella pyogenes*	1.29	0.84–1.99	0.245	0.327
Coinfection (≥2 pathogens)	1.62	0.59–4.50	0.350	0.438

**Table 7:** Multivariable ordinal logistic regression analysis for ultrasonographic severity score (SUlt).

Predictor	aOR	95% CI	*p*-Value (Raw)	*p*-Value (FDR-Adjusted)
*Mycoplasma ovipneumoniae*	1.91	1.22–2.97	**0.005**	0.050
*Pasteurella multocida*	2.58	1.55–4.31	**0.001**	**0.010**
*Mannheimia haemolytica*	1.86	1.07–3.25	**0.029**	0.116
*Trueperella pyogenes*	1.53	0.93–2.52	0.098	0.158
Coinfection (≥2 pathogens)	2.71	0.92–7.97	0.070	0.156

**Table 8:** Multivariable ordinal logistic regression analysis for post-mortem severity score (SPost).

Predictor	aOR	95% CI	*p*-Value (Raw)	*p*-Value (FDR-Adjusted)
*Mycoplasma ovipneumoniae*	1.64	1.09–2.48	**0.017**	0.113
*Pasteurella multocida*	1.50	0.99–2.28	0.057	0.156
*Mannheimia haemolytica*	1.19	0.75–1.87	0.464	0.516
*Trueperella pyogenes*	0.99	0.66–1.48	0.957	0.957
Coinfection (≥2 pathogens)	1.57	0.54–4.56	0.407	0.479

Footnote for Table 5, Table 6, Table 7 and Table 8: aOR, adjusted odds ratio; CI, confidence interval; FDR, false discovery rate. Adjusted odds ratios were estimated using multivariable proportional odds ordinal logistic regression models. Odds ratios > 1 indicate increased odds of belonging to a higher severity category (reference category: severity score = 0; pathogen not detected). All models simultaneously included *Mycoplasma ovipneumoniae*, *Pasteurella multocida*, *Mannheimia haemolytica*, *Trueperella pyogenes*, and coinfection status. Coinfection was defined as the detection of two or more respiratory pathogens in the same animal. Bold *p*-value (FDR-adjusted) values indicate statistical significance after Benjamini–Hochberg correction across the 20 individual tests.

**Table 9:** Distribution of severity scores (SClinic, SAusc, SUlt, SPost) stratified by SPCR Ct category for Mycoplasma ovipneumoniae (*n* = 80). Values are counts and percentages of animals in each severity category (S0, normal; S1, mild-moderate; S2, moderate-severe; S3, severe). The data correspond to Figure 5.

Outcome	Ct 0 (*n* = 36)	Ct 1 (*n* = 4)	Ct 2 (*n* = 12)	Ct 3 (*n* = 28)	*p*-Trend (Spearman)
SClinic: S0 (*n*)	17 (47.2%)	2 (50.0%)	3 (25.0%)	2 (7.1%)	ρ = +0.443, *p* < 0.001
SClinic: S1 (*n*)	14 (38.9%)	0 (0.0%)	5 (41.7%)	13 (46.4%)	
SClinic: S2 (*n*)	5 (13.9%)	2 (50.0%)	3 (25.0%)	7 (25.0%)	
SClinic: S3 (*n*)	0 (0.0%)	0 (0.0%)	1 (8.3%)	6 (21.4%)	
SClinic: mean ± SD	0.67 ± 0.76	1.00 ± 1.16	1.17 ± 0.94	1.61 ± 0.92	
SAusc: S0 (*n*)	21 (58.3%)	1 (25.0%)	5 (41.7%)	7 (25.0%)	ρ = +0.454, *p* < 0.001
SAusc: S1 (*n*)	10 (27.8%)	0 (0.0%)	1 (8.3%)	9 (32.1%)	
SAusc: S2 (*n*)	4 (11.1%)	1 (25.0%)	5 (41.7%)	7 (25.0%)	
SAusc: S3 (*n*)	1 (2.8%)	2 (50.0%)	1 (8.3%)	5 (17.9%)	
SAusc: mean ± SD	0.75 ± 0.97	1.75 ± 1.50	1.33 ± 1.23	1.71 ± 1.18	
SUlt: S0 (*n*)	19 (52.8%)	0 (0.0%)	1 (8.3%)	0 (0.0%)	ρ = +0.652, *p* < 0.001
SUlt: S1 (*n*)	10 (27.8%)	0 (0.0%)	0 (0.0%)	6 (21.4%)	
SUlt: S2 (*n*)	5 (13.9%)	2 (50.0%)	7 (58.3%)	5 (17.9%)	
SUlt: S3 (*n*)	2 (5.6%)	2 (50.0%)	4 (33.3%)	17 (60.7%)	
SUlt: mean ± SD	0.72 ± 0.97	2.50 ± 0.58	2.17 ± 0.94	2.39 ± 0.83	
SPost: S0 (*n*)	19 (52.8%)	0 (0.0%)	2 (16.7%)	3 (10.7%)	ρ = +0.471, *p* < 0.001
SPost: S1 (*n*)	7 (19.4%)	0 (0.0%)	3 (25.0%)	6 (21.4%)	
SPost: S2 (*n*)	8 (22.2%)	1 (25.0%)	3 (25.0%)	6 (21.4%)	
SPost: S3 (*n*)	2 (5.6%)	3 (75.0%)	4 (33.3%)	13 (46.4%)	
SPost: mean ± SD	0.81 ± 1.00	2.75 ± 0.50	1.75 ± 1.22	2.04 ± 1.07	

**Table 10:** Multivariable ordinal logistic regression analysis with SPCR Ct (ordinal 0–3) as the main predictor for *Mycoplasma ovipneumoniae*, adjusted for *Pasteurella multocida*, *Mannheimia haemolytica*, *Trueperella pyogenes*, and coinfection status (*n* = 80). The binary *Mycoplasma ovipneumoniae* variable was excluded because SPCR Ct ≥ 1 is collinear with *Mycoplasma ovipneumoniae* detection. aOR = adjusted odds ratio per one unit increase in SPCR Ct category; CI = confidence interval; raw *p* = unadjusted *p*-value; FDR *p* = Benjamini–Hochberg-adjusted *p*-value across the 4 outcomes.

Outcome	aOR (per Ct Unit)	95% CI	*p* (Raw)	*p* (FDR)
SClinic	1.43	0.97–2.10	0.070	0.070
SAusc	1.55	1.05–2.28	0.027	0.036
SUlt	1.98	1.30–3.01	0.0015	0.006
SPost	1.73	1.16–2.57	0.007	0.014

## Data Availability

The deidentified data presented in this study are available from the corresponding author upon reasonable request, subject to a data use agreement to protect farm-level privacy and commercial confidentiality.
